# Primary primitive neuroectodermal tumor of the orbit

**DOI:** 10.4103/0301-4738.55071

**Published:** 2009

**Authors:** Dipankar Das, Ganesh Chandra Kuri, Panna Deka, Kasturi Bhattacharjee, Harsha Bhattacharjee, Akshay C Deka

**Affiliations:** Sri Sankaradeva Nethralaya, Guwahati, Assam, India

**Keywords:** Histopathology, immunohistochemistry, primary primitive neuroectodermal tumor.

## Abstract

Primitive neuroectodermal tumor (PNET) is a small round cell malignant tumor of neuroectodermal origin. Most of the PNETs occur in the central nervous system (CNS). PNETs recognized outside of CNS are diagnosed as peripheral PNET (pPNET). This tumor which expresses MIC-2 gene (CD99) seems to be least aggressive after complete tumor resection. We describe a rare case of PNET in a young girl.

Peripheral primary primitive neuroectodermal tumor (PNET) is seen in young adults and adolescents and has no sex predilection. Primary orbital pPNET is extremely rare and only nine cases have been reported previously.[1–9] pPNET shows characteristic small round cell tumor with rosette or pseudo-rosette, positive immunohistochemistry (IHC) and in some cases there are ultrastructural findings of neurosecretory granules. This report presents the clinical, radiological and histopathologic features of a left orbital mass in a young girl. IHC confirmed the diagnosis of pPNET. This rare tumor of the orbit should be considered in the differential diagnosis of hypercellular small round cell tumor of the orbit.

## Case Report

**Figure 1:**
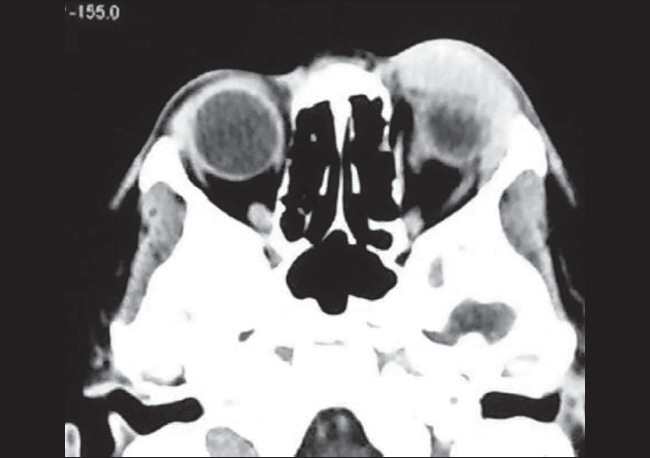
Computed tomography scan in axial view showing a soft tissue enhanced irregular mass in the antero-lateral aspect of the left orbit without evidence of bone erosion and defect

An eight-year-old female child was brought to our outpatient department for progressing protrusion of the left eye ball for two months. There was associated diplopia with restriction of ocular movements superiorly and laterally. There was minimal ptosis and she had ocular pain over a period of four weeks. There was no history of injury, fever, chronic cough or other systemic symptoms. Her ophthalmic examination did not reveal any additional pathology other than a localized mass in the anterior orbit. On examination, a tender, firm, fixed, non-pulsating globular mass (4 × 3 cm) with irregular surface was felt in the supero-temporal quadrant of the left orbit. Ocular examination was otherwise normal. Computed tomography (CT) scans of the orbit and brain depicted a soft tissue enhanced irregular mass located in the supero-temporal part of the left orbit without evidence of any bone defect or erosion [Fig. 1]. Complete hemogram, including general blood picture, erythrocyte sedimentation rate and X-ray chest were normal. Human immunovirus and HBsAg screening tests were negative. Anterior orbitotomy of the left orbit was done under general anesthesia and *en block* excisional biopsy of the tumor was performed. Grossly, the tumor was well encapsulated and measured 3.2 × 2.6 × 1.5 cm and weighed 12 g.

The 10% neutral buffered formalin-fixed tissue fragment was white and firm. It was embedded in paraffin and sections were stained with hematoxylin-eosin. Histopathological examination of the tumor revealed round cells having eosinophilic cytoplasm and round nuclei. The cells were arranged in sheets and cords with thin sclerotic stroma. Few pseudo-rosettes which were viable tumor cells around the blood vessels were seen. There was no evidence of necrosis and hemorrhage. [Fig. 2A] Sections were studied by IHC for neuron-specific enolase (NSE) (ab “BBS/NC/VI-HI4), leukocyte common antigen (LCA) CD45 (ab “2BII+PD 7/26”), S-100 protein (ab “S1/61/69”), Desmin (ab “D-33”) and MIC-2 (ab “12E7”) using avidin-biotin complex technique. These antibodies were applied synchronously with appropriate positive control slides. In our case, IHC was positive for MIC-2 gene (CD45-ve, S-100-ve, Desmin -ve, NSE-ve) [Fig. 2 B]. On the basis of these findings, a diagnosis of primary pPNET of the orbit was made. The patient's diplopia and pain resolved one week after the surgery. Complete systemic evaluation was carried out which was normal. Patient is being followed up every three months and orbital CT scans are repeated on every visit without any sign of recurrence. Patient is under close observation of oncologist and there are no signs of recurrence for the last six months.

**Figure 2A:**
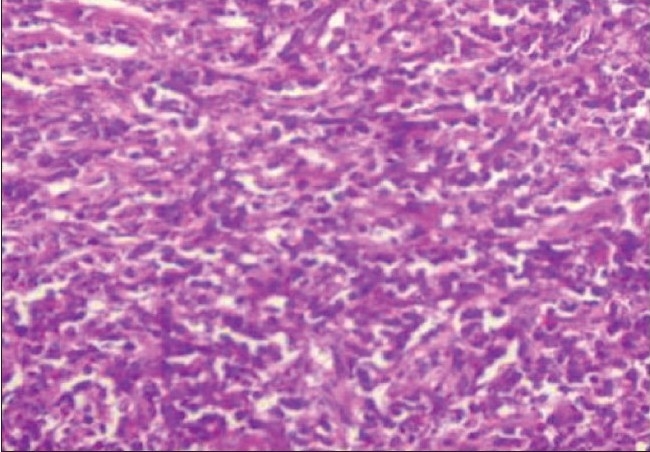
The tumor was composed of hypercellular, small round cells with hyperchromatic nuclei with occasional pseudo-rosettes (H&E, X200)

**Figure 2B:**
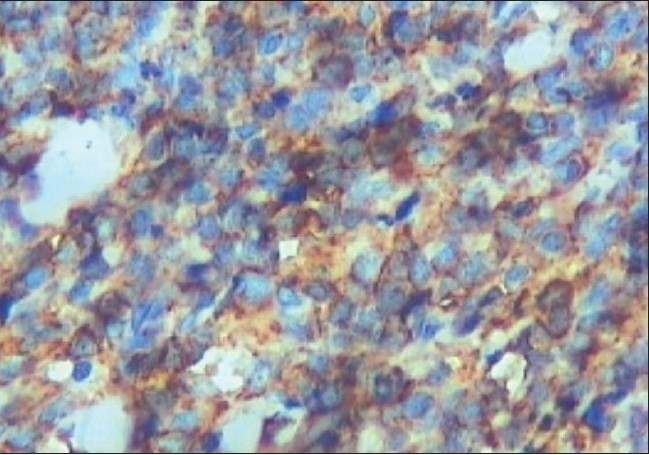
The tumor cells demonstrate MIC-2 positivity (the clone for “MIC-2” antibody is “12 E 7” original magnification X 400)

## Discussion

The differential diagnosis of pPNET of the orbit includes other small blue round cell tumors which include rhabdomyosarcoma (Actin+, Vimentin+, Desmin +, S-100-ve), Ewing's sarcoma (PGP9.5+, MIC-2+), lymphoma (LCA+, CD45+/CD20+/CD3+), neuroblastoma (PGP9.5+, MIC-2 -ve) and metastatic retinoblastoma to orbit (NSE+, GFAP+).[2–4] Peripheral primitive neuroectodermal tumors are rare lesions that form part of the Ewing family of tumors, which includes osseous and extraosseous Ewing's sarcoma. All are characterized by translocations involving the EWS gene at 22q12, usually the translocation t(11;22)(q24;12).[7,8] Histopathological appearances of a small, round, dark blue cell tumor with monotonous, highly cellular pattern, pseudo-rosette formation and strong membrane positivity for the MIC-2 gene product in IHC is the hallmark for diagnosis of pPNET. In some cases they are NSE, synaptophysin and S-100-positive.[7]

Alyahya *et al*.[8] had reported an orbital, intraconal pPNET in a five-year-old child with microphthalmia since birth. Orbitotomy was performed and a large, polycystic, retroscleral, intraconal tumor was removed. Subsequent histological, immunohistochemical and electron-microscopic analysis of the mass was performed. The tumor showed characteristic features of pPNET with pseudo-rosettes, positive IHC for the MIC-2 gene and synaptophysin and ultrastructral finding of neurosecretory granules. Their case report was the first orbital case of pPNET to express the MIC-2 gene.[8]

The MIC-2 gene is a pseudoautosomal gene located on the short arms of both X and Y chromosomes. The gene harvests glycoproteins of related molecular weight designated p30 and p32. The proteins programmed by MIC-2 gene are neuraminidase and protease sensitive. In red cells, MIC-2 gene is synchronized by the X-linked XG gene, resulting in quantitative polymorphism for level of MIC-2 gene product.[10]

MIC-2,12E7 is used in different dilutions determined on formalin-fixed paraffin-embedded tissue. A study of 70 different tumors has shown that among the neoplastic tissues, only glioblastoma and ependymoma of central nervous system (CNS) as well as certain islet cell tumors of pancreas reacted positively. Since the MIC-2 gene products are most strongly expressed on cell membranes of Ewing's sarcoma and pPNET, expression of the gene products allows for the differentiation of these tumors from other round cells tumors of childhood and adolescence.[10]

In this context, our case report is the second in literature where MIC-2 fraction of IHC was positive for pPNET. In spite of aggressive malignant features of pPNET, orbital variety seems to be the least aggressive since most of the reported patients are still alive.[8,10] In our case, we can better comment on the ultimate prognosis after we follow up the case for atleast two years.

The present case report highlights that differential diagnosis of hypercellular small round cell tumor of the orbit should be recognized and pPNET must be considered as possible diagnosis by the ocular pathologists. This rare tumor can be confirmed by IHC.
